# The mediating role of social capital in the relationship between socioeconomic status and adolescent wellbeing: evidence from Ghana

**DOI:** 10.1186/s12889-019-8142-x

**Published:** 2020-01-07

**Authors:** Evelyn Aboagye Addae

**Affiliations:** Department of Sociology and Social Policy, Lingnan University, 8 Castle Peak Road, Tuen Mun, Hong Kong

**Keywords:** Social capital, Socioeconomic status, Wellbeing, Sense of belonging, Autonomy and control, Life satisfaction, Happiness, Ghana, Social context

## Abstract

**Background:**

Social capital is generally portrayed to be protective of adolescents’ health and wellbeing against the effects of socioeconomic inequalities. However, few empirical evidence exist on this protective role of social capital regarding adolescents’ wellbeing in the low-and middle-income country (LMIC) context. This study examines the potential for social capital to be a protective health resource by investigating whether social capital can mediate the relationship between socioeconomic status (SES) and wellbeing of Ghanaian adolescents. It also examines how SES and social capital relate to different dimensions of adolescents’ wellbeing in different social contexts.

**Methods:**

The study employed a cross-sectional survey involving a randomly selected 2068 adolescents (13-18 years) from 15 schools (8 Senior and 7 Junior High Schools) in Ghana. Relationships were assessed using multivariate regression models.

**Results:**

Three measures of familial social capital (family sense of belonging, family autonomy support, and family control) were found to be important protective factors of both adolescents’ life satisfaction and happiness against the effects of socioeconomic status. One measure of school social capital (school sense of belonging) was found to augment adolescents’ wellbeing but played no mediating role in the SES-wellbeing relationship. A proportion of about 69 and 42% of the total effect of SES on happiness and life satisfaction were mediated by social capital respectively. Moreover, there were variations in how SES and social capital related to the different dimensions of adolescents’ wellbeing.

**Conclusion:**

Social capital is a significant mechanism through which SES impacts the wellbeing of adolescents. Social capital is a potential protective health resource that can be utilised by public health policy to promote adolescents’ wellbeing irrespective of socioeconomic inequalities. Moreover, the role of the family (home) in promoting adolescents’ wellbeing is superior to that of school which prompts targeted policy interventions. For a holistic assessment of adolescents’ subjective wellbeing, both life evaluations (life satisfaction) and positive emotions (happiness) should be assessed concomitantly.

## Background

The recognition of several elements of social capital as complements to public health strategies has become prominent in recent years as efforts are being made to understand and address the social and structural determinants of young people’s health and wellbeing [1–4]. Wellbeing is recognised as encompassing not only economic wellbeing but also emotional, psychological and social wellbeing, explicating the overall as “a positive state of mind and body, feeling safe and able to cope, with a sense of connection with people, communities and the wider environment” [5]. This definition comprises concepts of subjective and social wellbeing, psychosocial factors, and social capital.

Social capital, however, appears to embrace elements of both psychosocial factors and social wellbeing as it provides vital pathways through which the social environment can impact most dimensions of the wellbeing of adolescents [6, 7]. Nevertheless, social inequalities in adolescents’ wellbeing are often measured by examining disparities in adolescents’ socioeconomic status (SES) while social capital is largely overlooked. Socioeconomic status (SES) has been found to impact health and wellbeing directly [2, 8–11] and indirectly through psychosocial factors such as the poor’s inability to establish bonds and networks for their benefits [6, 7, 11]. These psychosocial factors, however, can function as protective mechanisms to offset some effects of SES inequalities on wellbeing. This is because studies have revealed that the psychosocial effects of SES can be mediated or attenuated by socioemotional or psychosocial resources accrued through social relationships within one’s social environment [6, 7, 11]. This, therefore, underpins the potential role of social capital as a protective health resource for, especially, adolescents from low affluence families. Social capital continues to be a very successful concept that has been conceptualised from several perspectives. Although Coleman, Bourdieu, and Putnam defined social capital from different perspectives [12–14], generally, Coleman, Bourdieu, and Putnam recognised social capital as a *valuable social asset* and as an *individual and community traits* that can be measured and assessed within a social network for health and wellbeing benefits. Two distinctions of the two conceptualisations (individual and collective- level) of social capital exist: cognitive (people’s perception about interpersonal relationships, reciprocity, and enforcement of group values) and structural social capital (number of social networks and structures of civic engagement) [15, 16]. Three distinctions: bonding (strong ties connecting family members, friends and colleagues), bridging (links between individuals/groups of different structural powers) and linking (links individuals and families to institutions to enhance their capacity to access resources) have been further identified based on the kinds of social ties available [17, 18]. To translate social capital to the wellbeing of adolescents, this study defines social capital as *a valuable social resource that can be accessed by adolescents at the individual and community level of their social contexts for promotions of health and wellbeing.* These resources can be assessed and accumulated from families, schools, peers, and neighbours to optimise health benefits and potentially protect adolescents’ wellbeing from the effects of socioeconomic inequalities [5–7, 11, 19]. As Coleman emphasised some negative facet of social capital [12], this present study recognises ‘high or excessive control’ within the family context as a negative facet of social capital that can negatively affect adolescents’ wellbeing.

Understanding the protective roles of social capital can hence aid endeavours to address adolescents’ wellbeing inequalities associated with SES especially in LMICs where many adolescents face risks associated with poverty and low SES. There is, however, less empirical evidence on the protective role of social capital in most LMICs. This present study, therefore, aims to investigate the potential for social capital to mediate the SES-wellbeing relationship of adolescents by providing empirical evidence from a LMIC-Ghana. This study again seeks to provide evidence on how SES and social capital relate to different dimensions of adolescents’ wellbeing in different social contexts for targeted policy recommendations. This study hypothesised that high levels of social capital would lead to high levels of wellbeing and social capital would mediate the relationship between SES and wellbeing.

## Methods

### Sample and design

This empirical analysis employed a school-based cross-sectional survey data obtained from an adolescent health and wellbeing project in 2018. The project was designed to investigate the role of socioeconomic status and social capital in adolescent health and wellbeing in Ghana. The UN and UNICEF defines adolescents as persons of age from 10 to 19 years. The adolescence period is further categorised into two stages, young adolescents (10-14 years) and older adolescents (15-19 years) [20]. A total of 2068 adolescents (13-18 years) were randomly selected through a multi-stage stratified sampling approach from 7 districts in the Upper West Region of Ghana. In the initial sampling stage, three sub-regional zones (low, medium and high poverty zone) were defined as primary sampling units based on their distinctive sociodemographic, sociocultural factors and poverty index. Using the Ghana poverty mapping, two districts were randomly selected from each of the low and high poverty incidence zones and three districts were selected from the medium poverty zone because it had the largest number of districts. This approach gave equal chances to all districts in the region to be chosen. For homogeneity in the sample, only mixed (boys and girls) public schools were selected because almost all the available Senior High Schools (SHS) in the region were public schools. In each district, one Junior High School (JHS) and one SHS were selected except for one district where two SHS were selected due to limited sample size making up eight SHS and seven JHS. In the schools, students were stratified and randomised proportionately based on the class sizes. Data were collected through questionnaires from qualified participants which was voluntary and anonymous by the researcher and a trained research assistant during appropriate school hours designated by the school authorities. The survey questionnaire was developed in English since English is the language used in Ghanaian schools. However, during the survey, further interpretation was given in the dialect used in the region to those who required it by the research assistant who was a native of the region. The survey on average lasted for about 45 min in each school and participants were compensated with stationaries for their time. This study was approved by the Committee on Human Research Publication and Ethics (CHRPE), School of Medical Sciences, Kwame Nkrumah University of Science and Technology and Komfo Anokye Teaching Hospital, Kumasi, Ghana (Ref: CHRPE/AP/542/18). Ethics approval was also granted by the Research Ethics Sub-Committee of Lingnan University, Hong Kong. Consent from the regional and district education directors and parents/guardians of the selected participants were also sought. Parents/guardians who would not permit their children to participate returned signed consent forms with their reasons to the researcher through their children. Study participants were fully briefed on the research purpose, anonymity, and confidentiality and also provided written consent to sign before the survey to ensure their autonomy in participating in the study.

### Dependent variables

Despite the clear differentiation between subjective wellbeing-life evaluations such as life satisfaction and positive emotions such as happiness, previous works on adolescent SES, social capital and wellbeing have focused mostly on life satisfaction whereas happiness has been mostly overlooked. This study, hence, employed two indicators of subjective wellbeing, namely, life satisfaction and happiness. Life satisfaction was measured using the Cantril ladder scale because it represents an important cognitive aspect of multidimensional wellbeing [21]. Respondents were shown an image of a ladder and requested to indicate a position on one of the 11 steps (10 the best possible life and 0 the worst life). Happiness was measured by the World-Value Survey 2010–2012 wave scale for happiness based on a single item [22]. ‘Taking all things together, would you say you are ….? ’ The response options were coded as 4 = ‘very happy’, 3 = ‘rather happy’, 2= ‘not very happy’, 1= ‘not happy at all’ and the ‘don’t know’ response was not scored.

### Independent variables

#### Socioeconomic status (SES)

Studies have revealed that accessing children’s SES with their parents’ SES is problematic because children fail to report correctly their parents’ income, educational level, and occupation, prompting the need for child-specific SES [23]. This study, therefore, adopted the 9-item material affluence scale (MAS) developed by Doku et al. [24] to specifically measure the SES of adolescents in Ghana. An 8-item scale was used in this present study because the remaining item (parents’ house ownership) reduced the reliability of the scale. The items included two broad categories: household assets (television, fridge, computer, radio, electricity, family car, and own room) and housing characteristics (blockhouse and non-block house). The household assets and housing characteristics were combined to form a composite variable representing the SES of the respondents, α = .703. Responses to having the listed household assets were coded as Yes = 1, No = 0 and living in a blocked house = 1, non-blocked house = 0, and ‘don’t know’ was not scored. The responses were scored and summed to obtain a composite score (0–8). The full items and coding of the MAS can be referred from Doku et al. [24].

#### Social capital

This present study adopted indicators from Morgan et al. [25] social capital framework to represent adolescents’ cognitive social capital. Two subdomains of social capital identified by Morgan et al. [26] to be protective health assets for young people’s wellbeing: “sense of belonging (identity and safety with their environments) and autonomy and control (perceptions of power to influence decisions)” were selected. A total of two indicators of social capital (family sense of belonging-FSB and family autonomy and control-FAC) were included in the family context, while one indicator (school sense of belonging-SSB) was included in the school context. In this present study’s analysis, FAC was separated into two composite indicators-family autonomy support (FAS) and family control (FC) based on recent literature’s claim that autonomy and control are two distinct constructs of parenting styles [27]. FAS and FC were hence measured using Marbelle & Grolnick [28] 18-item autonomy support scale (α = .87) and 9-item parental control scale (α = .73) which they designed specifically for assessing parental autonomy support and parental control respectively for children in Ghana and the United States. Responses for FAS and FC were coded as (1 = not true at all, 2 = not true, 3 = true, 4 = very true). The ‘don’t know’ response was not scored. The items for measuring FAS and FC can be referred from Marbelle & Grolnick [28]. The FSB scale was developed by combining three items adopted from the 4-item King and Boyd [29] family connectedness scale (the remaining item – ‘how much do you feel you want to leave home?’ reduced the reliability of the scale so was excluded) and one item adapted from the ‘International Society of Child Indicators (ISCI) [30] 12 yr olds Questionnaire’ to form a 4-item composite scale (α = .74). The items included: ‘how much do you feel your family understands you?’; ‘how much do you feel you and your family have fun together?’; ‘to what extent do you feel your family pays attention to you?’; and ‘how much do you feel safe at home?’. The responses included (1 = very little, 2 = somewhat, 3 = neutral, 4 = quite a bit, 5 = very much). The ‘don’t know’ response was not scored. The ‘SSB’ scale was developed by combining three items adopted from OECD’s [31] six item-PISA SSB scale (the remaining 3 items reduced the reliability of the scale so were excluded) and other three items adapted from Morgan et al. [25] social capital framework forming a 6-item scale (α = .72): ‘I feel like I belong at school’; ‘ I make friends easily at school’; ‘other students seem to like me’; ‘most of the students in my class (es) are kind and helpful’; ‘if I have a problem at school my teachers will help me’; and ‘my teachers care about me’. Responses were coded as (5 = strongly agree, 4 = agree, 3 = neutral, 2 = disagree, 1 = strongly disagree). A number of items were therefore employed to create composite indicators representing the respondents’ social capital (FSB, SSB, FAS, and FC). The responses were scored and summed up to obtain a composite score. For example; the respondents’ FSB comprised of 4 statements such as ‘how much do you feel your family understands you?’ There were five possible responses excluding the ‘don’t know’ response, and these were scored as follows: very little (1); somewhat (2); neutral (3); quite a bit (4); and very much (5). The composite scores hence ranged from 4 to 20.

#### Sociodemographic characteristics (SDCs)

The role of 3 categories of SDCs: personal (age, gender, self-rated health, marital status, religion, and ethnicity), family (number of siblings and family structure) and school characteristics (class level, school residency, district, and bullying status) were also considered in the analyses due to their possible influence on in-school adolescents’ wellbeing. This was to ensure that a broad sample of sociodemographic characteristics that are mostly ignored in adolescent wellbeing studies was controlled for in the analysis to confirm the robustness of the mediating role of social capital in the SES-wellbeing relationship. The respondents’ age ranged from 13 to 18 years and were collapsed into two groups-young adolescents (13-14 yrs) and older adolescents (15-18 yrs). The class level was categorised into secondary (SHS 1 and SHS 2) and basic education level (JHS and JHS 2). All measures of the SDCs can be found in Table 1.

**Table 1:** Descriptive analysis of in-school adolescents employed in the study

	Valid N	(%)	Mean	(SD)	Range
Variables					
Personal Characteristics					
Age			16.25	(±1.492)	13–18
Age Cohort					
Young adolescent	600	(29.01)			
Older adolescent	1468	(70.99)			
Gender					
Male	988	(47.8)			
Female	1080	(52.2)			
Ethnicity					
Mole	23	(1.1)			
Dagbon	94	(4.5)			
Grusi	21	(1.0)			
Lobi	50	(2.4)			
Dagaaba or Dagaate	1252	(60.5)			
Sissala	96	(4.6)			
Waala	266	(12.9)			
Brifor	148	(7.2)			
Other	118	(5.7)			
Marital status					
Never married	1905	(92.1)			
Married	65	(3.1)			
Separated / broke-up	18	(0.9)			
Cohabiting	65	(3.1)			
Other	15	(0.7)			
Religious affiliation					
Christian	1501	(72.6)			
Muslim	548	(26.5)			
Traditionalist	19	(0.9)			
Self-rated health					
Low	575	(27.8)			
High	1493	(72.2)			
School characteristics					
District					
Nadowli-kaleo	300	(14.5)			
Wa west	299	(14.5)			
Wa Municipal	300	(14.5)			
Jirapa	298	(14.4)			
Lawra	300	(14.5)			
Daffiama	298	(14.4)			
Wa East	273	(13.2)			
School residential status					
Day Student	789	(38.2)			
Boarder	1279	(61.8)			
Class level					
JHS 1	380	(18.4)			
JHS 2	294	(14.2)			
SHS 1	956	(46.2)			
SHS 2	438	(21.2)			
Bullying status					
Yes	797	(38.6)			
No	1266	(61.4)			
Family characteristics					
Family structure					
Single Parent	417	(20.2)			
Both Parents	1262	(61.0)			
Stepparents	108	(5.2)			
Family relatives	271	(13.1)			
Other	10	(0.5)			
Number of siblings					
No siblings	46	(2.2)			
1-3siblings	592	(28.6)			
4-6siblings	1082	(52.3)			
7-10siblings	324	(15.7)			
Above 10	24	(1.2)			

*N* = Sample size*, % =* sample percentage*, SD* Standard deviation

### Data analysis

Univariate analysis using descriptive statistics involving all the sociodemographic characteristics was done and their frequencies, mean, standard deviation and percentages were derived. Spearman correlation between dependent and independent variables was also carried out to test the initial hypotheses of the relationships among SES, social capital and wellbeing for further analysis. Multivariate analyses using Model 4 in SPSS-PROCESS Macro Hayes’s version 3.3 was done to determine the hypothesised relationship among SES, social capital and wellbeing and the mediating role of social capital in the SES-wellbeing relationship while controlling for all the sociodemographic variables. PROCESS employs a path analytical framework and a bootstrapping approach to deliver powerful estimates of direct and indirect effects. The indirect effect was tested using a bootstrapping estimation approach with a bootstrap sample of 5000 at a 95% bias-corrected confidence interval (95%BCCI). The bootstrapping technique employed a random resampling process to approximate statistics on a sample by sampling the available dataset with 5000 replacements. The bootstrapping technique offers an efficient means to guarantee that the models employed are constant and reliable for the analysis to produce more precise results. It allows inferences about indirect effects to be made. The endpoints of the confidence interval are defined by percentiles in the allocation of bootstrap estimates of the indirect effect [32]. The mediating effects of the supposed mediators (FSB, SSB, FAS, and FC) were, hence, confirmed by using the lower and upper limit values of the confidence intervals. The assumption is that the interval between the lower and upper limits should not contain 0 for the mediation to be significant (both confidence intervals should either be positive or negative). The bootstrapping technique was analysed in two separate models-Model 1 and Model 2. Model 1 examined the mediating role of social capital between the SES-life satisfaction relationship while Model 2 examined the mediating role of social capital between the SES-happiness relationship. All analyses in this study were conducted using IBM-SPSS for Windows application (version 23.0) software and the level of significance was *p* < 0.05 (two-tailed).

## Results

### Sample characteristics

Table 1 contains the descriptive statistics of all the variables used in the study. There were more females (*n* = 1080) than males (*n* = 988); the mean age of the respondents was 16 yrs and age was categorised into young (*n* = 600) and older adolescents (*n* = 1468); about 8% of the sample were either married, married but separated and co-habiting; about 28% reported low self-rated health; about 72% were Christians and about 5% were non-natives of the study region. The majority were living with both biological parents (61%) and about 2.2% had no siblings. There were more secondary level students (*n* = 1279) than basic level students (*n* = 789); about 27% were from schools located in the two districts with the highest poverty index in the region (Wa West and Wa East); about 62% were residing on school campuses (boarders) and 39% reported been bullied in the past 2 months period to the study survey.

### Bivariate results

The Spearman correlation analyses revealed significant positive associations between SES and life satisfaction (*r* = .173, *p* < 0.001) and happiness (*r* = .117, *p* < 0.001) (Table 2). The analyses also showed that those with high family sense of belonging (FSB) (*r* = .409, *p* < 0.001), high school sense of belonging (SSB) (*r* = .192, *p* < 0.001), high family autonomy support (FAS) (*r* = .331, *p* < 0.001) and low family control (FC) (*r* = −.214, *p* < 0.001) are more likely to have high life satisfaction. Furthermore, FSB (*r* = .409, *p* < 0.001), SSB (*r* = .194, *p* < 0.001), and FAS (*r* = .370, *p* < 0.001) showed significant positive associations with happiness whiles, FC showed a negative association with happiness, FC (*r* = −.117, *p* < 0.001). The correlation results support the initial hypotheses that SES and social capital are related and are also related to wellbeing. (see Table 2). Although the correlations between most of the variables are highly significant, most of the correlation coefficients are below 0.4 positing that the correlations between the respondents’ SES, social capital, life satisfaction and happiness were not strong.

**Table 2:** SPSS-Spearman correlation matrix among socioeconomic status, social capital, life satisfaction, and happiness of in-school adolescents

	N	1	2	3	4	5	6	7
1. Life satisfaction	2068							
2. Happiness	2068	.424^***^						
3. Family sense of belonging	1909	.409^***^	.409^***^	**(.744)**				
4. school sense of belonging	2068	.192^***^	.194^***^	.238^***^	**(.716)**			
5. Family autonomy support	1479	.331^***^	.370^***^	.407^***^	.185^***^	**(.874)**		
6. Family control	1730	−.214^***^	−.117^***^	−.127^***^	−.004	−.041	**(.728)**	
7. Socioeconomic status	2068	.173^***^	.117^***^	.170^***^	.056^*^	.144^***^	−.104^***^	**(.703)**

*** *p* < 0.001, *p < 0.05, *N* Sample size, Cronbach’s alphas of scales employed in this study are shown on the diagonal bold in brackets

### Multivariate relationships

#### Life satisfaction

##### The direct relationship between socioeconomic status, social capital and, life satisfaction

**Fig. 1:**
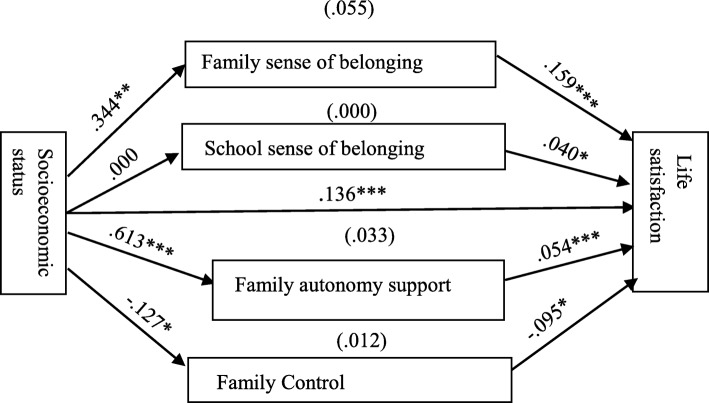
Predicted relationships among socioeconomic status, social capital and life satisfaction. Paths estimates in Model 1 (*N* = 1, 236; ***p < .001, **p < .005 *p < .05)

As shown in Table 3 and Fig. 1, the results supported the findings from the correlation matrix and the hypothesis that those with high SES and high social capital are more likely to have high life satisfaction than those with low SES and low social capital. SES significantly predicted life satisfaction (*B* = 0.136, *p* < .001); family sense of belonging (FSB) (*B* = 0.344, *p* < .005); family autonomy support (FAS) (*B* = 0.613, *p* < .001); and family control (FC) (*B* = − 0.127, *p* < .05) but not school sense of belonging (SSB) (*B* = 0.000). Also, FSB (*B* = 0.159, *p* < .001); SSB (*B* = 0.040, *p* < .05); FAS (*B* = 0.054, *p* < .001) and FC (*B* = − 0.095, *p* < .001) significantly predicted life satisfaction (see Table 3 and Fig. 1).

**Table 3:** Results of the direct effect of socioeconomic status and social capital on the life satisfaction of in-school adolescents: Model 1

Predictors	CE (*B*)	SE	*t*	*p*
Socioeconomic status	0.136	0.037	3.709	<.001
Family sense of belonging	0.159	0.019	8.561	<.001
School sense of belonging	0.040	0.017	2.383	<.05
Family autonomy support	0.054	0.009	5.823	<.001
Family control	−0.095	0.017	−5.458	<.001
Total effect				
Total effect of Socioeconomic status on life satisfaction	0.236	0.039	0.040	<.001

*N* = 1236, *B* Unstandardised coefficients, *SE* Standard error, *t* T-test. All the sociodemographic variables were controlled for in the model

##### The indirect effect of socioeconomic status on life satisfaction through social capital

Table 4 and Fig. 1 show that the effect of SES on life satisfaction was mediated by social capital (FSB, FAS, and FC) in Model 1, except that SSB played no mediating role (because the confidence intervals derived contained 0). In Model 1, about 23% proportion of the total effect of SES on life satisfaction was mediated by FSB (*B* = 0.055, 95% CI [0.033, 0.079]) while about 14% proportion was mediated by FAS (*B* = 0.033, 95% CI [0.017, 0.053] and about 5% proportion was mediated by FC (*B* = 0.012, 95% CI [0.002, 0.025]. SSB did not mediate the effect of SES on life satisfaction. A total proportion of about 42% of the total effect of SES on life satisfaction was therefore mediated by social capital.

**Table 4:** Result of bootstrapping mediation analysis in Model 1: Assessing social capital as a mediator in the relationship between socioeconomic status (SES) and life satisfaction

Indirect Path	Indirect Effect		95% BCCI (*B*)		Ratio (*100) Specific Mediation Effect to Total Effect^a^ (%)
	*B*	BootSE	Lower	Upper	
SES → FSB → LS	0.055	0.012	0.033	0.079	23.305
SES → SSB → LS	0.000	0.003	− 0.006	0.006	0.000
SES → FAS → LS	0.033	0.009	0.017	0.053	13.983
SES → FC → LS	0.012	0.006	0.002	0.025	5.085

*N* = 1236, *B* Unstandardised coefficients, *BootSE* Bootstrapping standard error, *BCCI* Bias-corrected confidence intervals, ^a^ Ratio calculated as 100 × (indirect effect (*B)* / total effect), where the total effect is the sum of all mediation effects (i.e., the sum of indirect effects) and the direct effect [33]. About a total proportion of 42.38% was mediated by social capital. All the sociodemographic variables were controlled for in the model

#### Happiness

##### The direct relationship between socioeconomic status, social capital and, happiness

**Fig. 2:**
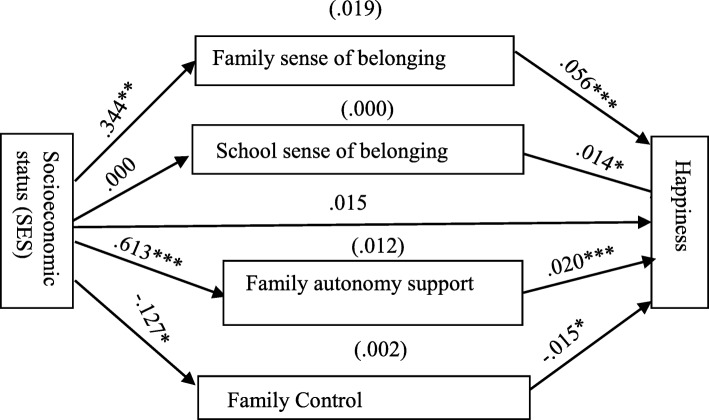
Predicted relationships among socioeconomic status, social capital and happiness. Paths estimates in Model 2 (*N* = 1, 236; ***p < .001, **p < .005, *p < .05)

As shown in Table 5 and Fig. 2, there is a positive relationship between SES and family sense of belonging (FSB), family autonomy support (FAS), and school sense of belonging (SSB) but a negative relationship between SES and family control (FC). Although the correlation result shows a positive relationship between SES and happiness, SES could not significantly predict happiness in the mediation model-Model 2 when social capital and the sociodemographic variables were controlled for. This is contrary to the hypothesis that those with high SES are more likely to have high happiness. SES significantly predicted FSB (*B* = 0.344, *p* < .005); FAS (*B* = 0.613, *p* < .001); and FC (*B* = − 0.127, *p* < .05); but not SSB (*B* = 0.000) as shown in Fig. 2. Again, social capital: FSB (*B* = 0.056, *p* < .001); SSB (*B* = 0.014, *p* < .05); FAS (*B* = 0.020, *p* < .001); and FC (*B* = − 0.015, *p* < .05) significantly predicted happiness in Model 2. (see Table 5 and Fig. 2).

**Table 5:** Results of the direct effect of socioeconomic status and social capital on the happiness of in-school adolescents: Model 2

Predictors	CE (*B*)	SE	*t*	*p*
Socioeconomic status	0.015	0.012	1.196	.232
Family sense of belonging	0.056	0.006	8.988	<.001
School sense of belonging	0.014	0.006	2.512	<.05
Family autonomy support	0.020	0.003	6.378	<.001
Family control	−0.015	0.006	−2.539	<.05
Total effect				
Total effect of socioeconomic status on happiness	.048	.013	3.677	<.001

*N* = 1236, *B* Unstandardised coefficients, *SE* Standard error, *t* T-test. All the sociodemographic variables were controlled for in the model

##### The indirect effect of socioeconomic status (SES) on happiness through social capital

As shown in Table 6, in Model 2, the effect of SES on happiness was mediated by social capital. While family sense of belonging (FSB), family autonomy support (FAS), and family control (FC) played significant mediating roles between the SES-happiness relationship (because all their confidence intervals do not contain 0), school sense of belonging (SSB) played no mediating role. About 40% proportion of the total effect of SES on happiness was mediated by FSB (*B* = 0.019, 95% CI [0.012, 0.028]), while about 25% proportion was mediated by FAS (*B* = 0.012, 95% CI [0.006, 0.019]) and about 4% proportion was mediated by FC (*B* = 0.002, 95% CI [0.000, 0.004]). SSB did not mediate the SES-happiness relationship. About a total proportion of 69% of the total effect of SES on happiness was hence mediated by social capital in Model 2.

**Table 6:** Result of bootstrapping mediation analysis in Model 2: Assessing social capital as a mediator in the relationship between socioeconomic (SES) and happiness

Indirect Path	Indirect Effect		95% BCCIs (*B*)		Ratio (*100) Specific Mediation Effect to Total Effect^a^ (%)
	*B*	BootSE	Lower	Upper	
SES → FSB → LS	0.019	0.004	0.012	0.028	39.583
SES → SSB → LS	0.000	0.001	−0.002	0.002	0.000
SES → FAS → LS	0.012	0.003	0.006	0.019	25.000
SES → FC → LS	0.002	0.001	0.000	0.004	4.167

*N* = 1236, *B* Unstandardised coefficients, *BootSE* Bootstrapping standard error, *BCCIs* Bias-corrected confidence intervals, ^a^ Ratio calculated as 100 × (indirect effect (*B)* / total effect), where the total effect is the sum of all mediation effects (i.e., the sum of indirect effects) and the direct effect [33]. About a total proportion of 69% was mediated by social capital. All the sociodemographic variables were controlled for in the model

## Discussion

The positive relationship between SES, social capital and wellbeing have been shown by many studies. However, this important relationship has not been accounted for with respect to adolescents’ life satisfaction and happiness concurrently, a major strength of the present study. This approach has provided a different perspective for understanding how SES and social capital are related to different dimensions of adolescents' wellbeing within different social contexts.

This study found that adolescents with high SES and high social capital stand greater chances of achieving high life satisfaction than those with low SES and low social capital after controlling for all sociodemographic variables corroborating the observations of some previous studies [2, 6–9, 11]. The positive relationship between SES and life satisfaction could be related to the material effect of SES on health [2]. Thus, the respondents with low SES might have reported low life satisfaction possibly because of the direct physiological effects of lack of financial and material resources, which may inform, for example, poor shelter and inadequate material assets considering that the study participants were selected from the poorest region in Ghana. The finding that those with high social capital have high life satisfaction could also be linked to the socioemotional support derived from a sense of belonging, autonomy support, and control [34, 35]. It was again found in this study that those with high social capital were more likely to feel happier compared to those with low social capital even after adjustments. On the other hand, the level of the respondents’ SES did not relate to happiness when social capital and the sociodemographic variables were considered, reflecting a possible psychosocial effect of SES through social capital [6, 7, 11, 36–38].

These findings on the direct effect of SES and social capital on wellbeing is supported by Diner et al. [36] and Sengupta et al. [37] who found that SES is more related to life satisfaction than happiness and that the indicators of social capital including social-psychological needs for a sense of belonging and autonomy are more related to happiness as compared to life satisfaction. Similarly, Puntscher et al. [38] found family and friendship ties to be positively related to happiness but not life satisfaction. It is, hence, probable that the respondents assessed their life satisfaction based on their material conditions while they assessed their happiness based on their psychosocial conditions-social capital. The positive relationship between social capital and happiness can also be linked to the psychological benefits such as self-esteem and self-worth which is derived from a sense of belonging, autonomy, and control [34, 35]. This study, moreover, found that autonomy support and control are crucial for the wellbeing of adolescents in a collectivist society-Ghana [28, 34]. Additionally, similar to assertions that parental autonomy support and control are two distinct constructs of parenting styles [27], the present study indicates that there are variations in how autonomy and control relate to different dimensions of wellbeing and the effect of family control on the wellbeing of Ghanaian adolescents is secondary to that of family autonomy support.

More importantly, this study expands knowledge on the protective role of social capital amidst SES inequalities. As revealed by other studies [6, 7, 11, 36–38], this present study found that even in the presence of SES, the impact of social capital on wellbeing is still vital. Thus, adolescents who are from poor households but have high level of social capital are more likely to be protected from wellbeing inequalities compared to adolescents who are from poor households and also have low level of social capital. Family sense of belonging, family autonomy support, and family control mediated the effects of SES on both the life satisfaction and happiness of the respondents in support of other studies globally. However, social capital presented a stronger mediation in the SES-happiness relationship compared to its role in the SES-life satisfaction relationship. This confirms why the direct effect of SES on happiness was not significant when social capital was controlled for but was significant for life satisfaction.

High sense of belonging and autonomy support have been found to increase self-worth, intrinsic motivation, and perceived meaningfulness in life to some extent [34, 39] which can possibly boost high optimism for the future irrespective of one’s current poor conditions. This may likely explain why irrespective of the generally low SES of the respondents, overall, they reported high wellbeing. These findings also indicate that adolescents are indirectly affected by SES through the impacts of their families’ behaviour towards them (sense of belonging, autonomy support and control). Thus, adolescents from low SES households are more likely to have parents who employ power-assertive discipline and tend to show less socioemotional support for their children compared to adolescents from high SES households. Consequently, adolescents from low SES families tend to have poor wellbeing.

On the other hand, school sense of belonging could not mediate both the SES-life satisfaction and SES-happiness relationships. The significant relationship between SES and school sense of belonging implies that SES is important for promoting the SSB of in-school adolescents. Adolescents from high SES families are, hence, possibly protected from having low school sense of belonging. However, when the other measures of social capital and the sociodemographic variables were controlled for in the mediation models, SES could not significantly predict school sense of belonging. The inability for school sense of belonging to mediate the SES-wellbeing relationship implies that, even in the presence of school sense of belonging, the effects of SES on wellbeing can still be detrimental [31].

These findings on the mediating role of social capital infer that the family context (home) is more protective of adolescents’ wellbeing than other social contexts (school). Thus, only family sense of belonging, family autonomy support, and family control were protective of both the respondents’ life satisfaction and happiness. Also, considering that only 42 and 69% proportion of the total effect of SES on life satisfaction and happiness were mediated in Model 1 and Model 2 respectively infers that the sociodemographic variables also played a part in the effect of SES on the respondents’ wellbeing outcomes. This implies that sociodemographic factors can partly affect the relationship between SES, social capital and wellbeing.

### Strengths and limitations

This study has some strengths. Firstly, this study employed sub-domains of social capital that were developed as part of the World Health Organisation-Health Behaviour in School-aged Children (WHO-HBSC) optional package and have been widely validated and confirmed in cross-national studies by researchers to be protective health assets for young people [7]. Employing these sub-domains of social capital provides strong bases for social and public health practitioners to acknowledge the significance of the findings from this study. Secondly, this study employed validated and reliable measurement scales that were developed purposely for adolescents in LMICs-Ghana as well as scales validated to be homogeneous. This adds more confidence to the representation of the SES, social capital and wellbeing of adolescents in Ghana. Thirdly, this study is one of the few to investigate the interplay among adolescents’ SES, social capital and life satisfaction and happiness concurrently in the LMIC context. This study can hence set the pace for other researchers interested in promoting social capital and wellbeing of young people in LMICs.

The limitation of this study is that using cross-sectional data did not allow any causality to be established between SES, social capital and wellbeing. Secondly, although representative, only a sample from Northern Ghana was employed in the study. Considering the two distinct regional socioeconomic positions in Ghana, a sample constituting both adolescents from Northern and Southern Ghana would have helped to provide a more robust generalisation of the findings. A future study should hence include survey data from both regions.

## Conclusions

This study emphasises that the social environment can offer itself to a healthy, satisfactory, and happy life for adolescents from low SES families in LMICs irrespective of their contextual and sociodemographic characteristics. This study has provided some evidence to suggest that social capital is more important for Ghanaian adolescents’ wellbeing outcomes than their SES. The effects of SES on adolescents' wellbeing would be more detrimental in the absence of social capital to mediate the SES-wellbeing relationship.

Sense of belonging, autonomy support and control obtained within the family and school contexts are accentuated as significant factors that can alter the wellbeing of Ghanaian adolescents. Considering the significant protective role of family sense of belonging, family autonomy support and family control in this study, familial social capital is a potential principal protective health resource and a principal *socioeconomic risk absorber (mediator)* for adolescents’ wellbeing outcomes compared to other social contexts (school). Thus, although a school sense of belonging is vital for high wellbeing, it does not function as a protective resource against the effects of SES on the wellbeing of Ghanaian adolescents. This reveals significant policy implications for the educational sectors to also acknowledge students’ socioeconomic circumstances in schools' health and wellbeing strategies. More importantly, this study supports the protective role of social capital for adolescents and reveals the need for the family and school contexts to be crucial targets for policy and intervention strategies targeting the subjective wellbeing and social capital of especially, low affluent adolescents. Public health strategies employing social approaches to health should, hence, adopt an integrated approach with families and school authorities.

Finally, this study has underlined the significant variations in the effect of SES and social capital on the life satisfaction and happiness of adolescents from a LMIC context by revealing that unlike social capital, high SES does not necessarily promise high happiness. It is hence vital for health researchers to promote holistic subjective wellbeing assessment by addressing both life evaluations (life satisfaction) and positive emotions (happiness) as they are affected by different conditions of adolescents’ life.

## Data Availability

The datasets used and/or analysed during the current study are available from the corresponding author on reasonable request.

## Abbreviations

FAC: Family autonomy and control
FAS: Family autonomy support
FC: Family control
FSB: Family sense of belonging
JHS: Junior High School
LMIC: Low-and middle-income country
LMICs: Low-and middle-income countries
SES: Socioeconomic status
SHS: Senior High School
SSB: School sense of belonging

## References

[1] Bwalya JC, Sukumar P (2019). The Relationship Between Social Capital and Children’s Health Behaviour in Ireland. Available at SSRN 3418320.

[2] Inchley J, Currie D (2016). Growing up unequal: gender and socioeconomic differences in young People’s health and wellbeing: health behaviour in school-aged children (HBSC) study. Health policy for children and adolescents No.

[3] Viner R (2017). How to measure enabling and supportive Systems for Adolescent Health.

[4] Klocke A, Stadtmüller S (2019). Social capital in the health development of children. Child Indic Res.

[5] Morgan A, Rivera F, Moreno C, Haglund BJA (2012). Does social capital travel? Influences on the life satisfaction of young people living in England and Spain. BMC Public Health.

[6] Buijs T, Maes L, Salonna F, Van Damme J, Hublet A, Kebza V, Costongs C, Currie C, De Clercq B (2016). The role of community social capital in the relationship between socioeconomic status and adolescent life satisfaction: mediating or moderating? Evidence from Czech data. Int J Equity Health.

[7] Ge T. Effect of socioeconomic status on children’s psychological wellbeing in China: the mediating role of family social capital. J Health Psychol. 2018:1-10. 10.1177/1359105317750462. 10.1177/1359105317750462 29278935

[8] Moreno-Maldonado C, Ramos P, Moreno C, Rivera F (2019). Direct and indirect influences of objective socioeconomic position on adolescent health: the mediating roles of subjective socioeconomic status and lifestyles. Int J Environ Res Public Health.

[9] Elgar FJ, McKinnon B, Torsheim T, Schnohr CW, Mazur J, Cavallo F, Currie C (2016). Patterns of socioeconomic inequality in adolescent health differ according to the measure of socioeconomic position. Soc Indic Res.

[10] Xu F, Cui W, Xing T, Parkinson M (2019). Family socioeconomic status and adolescent depressive symptoms in a Chinese low–and middle–income sample: the indirect effects of maternal care and adolescent sense of coherence. Front Psychol.

[11] Moore GF, Littlecott HJ, Evans R, Murphy S, Hewitt G, Fletcher A (2017). School composition, school culture and socioeconomic inequalities in young people's health: multi-level analysis of the health behaviour in school-aged children (HBSC) survey in Wales. Br Educ Res J.

[12] Coleman JS (1988). Social capital in the creation of human capital. Am J Sociol.

[13] Bourdieu P. The forms of capital. In: Richardson JG, editor. Handbook of theory and research for the sociology of education. New York: Greenwood; 1986. p. 241-58.

[14] Putnam RD (2000). Bowling alone: the collapse and revival of American community.

[15] Baum FE, Ziersch AM (2003). Social capital. J Epidemiol Community Health.

[16] Harpham T (2008). The measurement of community social capital through surveys. Social capital and health.

[17] Kim D, Subramanian SV, Kawachi I (2008). Social capital and physical health. Social capital and health.

[18] Szreter S, Woolcock M (2004). Health by association? Social capital, social theory, and the political economy of public health. Int J Epidemiol.

[19] Novak D, Emeljanovas A, Mieziene B, Štefan L, Kawachi I (2018). How different contexts of social capital are associated with self-rated health among Lithuanian high-school students. Glob Health Action.

[20] Camilletti E (2018). Realizing an enabling environment for adolescent well-being: an inventory of laws and policies for adolescents in South Asia.

[21] Cantril H (1965). The pattern of human cancer. Rutgers University press; 1965.

[22] Inglehart R, Haerpfer C, Moreno A, Welzel C, Kizilova K, Diez-Medrano J, Lagos M, Norris P, Ponarin E, Puranen B (2014). World Values Survey: Round Six-Country-Pooled Datafile 2010-2014.

[23] Currie CE, Elton RA, Todd J, Platt S (1997). Indicators of socioeconomic status for adolescents: the WHO health behaviour in school-aged children survey. Health Educ Res.

[24] Doku D, Koivusilta L, Rimpelä A (2010). Indicators for measuring material affluence of adolescents in health inequality research in developing countries. Child Indic Res.

[25] Morgan A, Rivera F, Moreno C, Haughland BJA (2012). (2012). Does social capital travel? Influences on the life satisfaction of young people living in England and Spain. BMC Public Health.

[26] Morgan A, Rivera F, Moreno C, Haglund BJA (2012). Does social capital travel? Influences on the life satisfaction of young people living in England and Spain. BMC Public Health.

[27] Hauser Kunz J, Grych JH (2013). Parental psychological control and autonomy granting: distinctions and associations with child and family functioning. Parenting..

[28] Marbell KN, Grolnick WS (2013). Correlates of parental control and autonomy support in an interdependent culture: a look at Ghana. Motiv Emot.

[29] King V, Boyd LM (2016). Factors associated with perceptions of family belonging among adolescents. J Marriage Fam.

[30] ISCI (2013). International society for child indicators (ISCI) (2013) 12 years-old questionnaire. Update no. 7.

[31] OECD. PISA 2015 results (Volume III): students’ well-Being, PISA. OECD Publishing: Paris; 2017. 10.1787/9789264273856-en. Accessed Feb 2018.

[32] The PROCESS macro for SPSS and SAS. https://processmacro.org/index.html. Accessed 11 September 2019.

[33] Mascha EJ, Dalton JE, Kurz A, Saager L (2013). Understanding the mechanism: mediation analysis in randomized and nonrandomized studies. Anesth Analg.

[34] Marbell-Pierre KN, Grolnick WS, Stewart AL, Raftery-Helmer JN (2019). Parental autonomy support in two cultures: the moderating effects of adolescents’ self-Construals. Child Dev.

[35] Di Domenico SI, Fournier MA (2014). Socioeconomic status, income inequality, and health complaints: a basic psychological needs perspective. Soc Indic Res.

[36] Diener E, Ng W, Harter J, Arora R (2010). Wealth and happiness across the world: material prosperity predicts life evaluation, whereas psychosocial prosperity predicts positive feeling. J Pers Soc Psychol.

[37] Sengupta NK, Osborne D, Houkamau CA, Hoverd WJ, Wilson MS, Halliday L, West-Newman T, Barlow FK, Armstrong G, Robertson A, Sibley CG (2012). How much happiness does money buy? Income and subjective wellbeing in New Zealand. N Z J Psychol.

[38] Puntscher S, Hauser C, Walde J, Tappeiner G (2015). The impact of social capital on subjective well-being: a regional perspective. J Happiness Stud.

[39] Lambert NM, Stillman TF, Hicks JA, Kamble S, Baumeister RF, Fincham FD (2013). To belong is to matter: sense of belonging enhances meaning in life. Personal Soc Psychol Bull.

